# Comparison of COVID-19 Vaccine Policies in Italy, India, and South Africa

**DOI:** 10.3390/vaccines10091554

**Published:** 2022-09-18

**Authors:** Manfei Yang, Leiyu Shi, Haiqian Chen, Xiaohan Wang, Jun Jiao, Meiheng Liu, Junyan Yang, Gang Sun

**Affiliations:** 1 Department of Health Management, School of Health Management, Southern Medical University, Guangzhou 510515, China; 2 Department of Health Policy and Management, Bloomberg School of Public Health, Johns Hopkins University, Baltimore, MD 21205, USA

**Keywords:** COVID-19, vaccine policies, vaccination, effective reproduction rate

## Abstract

(1) Purpose: This study aimed to analyze coronavirus disease 2019 (COVID-19) vaccine policies and their effectiveness in Italy, India, and South Africa to provide empirical experience for vaccination and COVID-19 pandemic control. (2) Methods: The study systematically summarized the COVID-19 vaccine policies in Italy, India, and South Africa through public information available on the official websites of the World Health Organization and the ministries of health in these three countries. Total vaccinations, COVID-19 vaccination rates, rates of fully vaccinated, rates of booster-vaccinated, and total confirmed cases were selected for cross-sectional comparison of COVID-19 vaccination in these three countries. Daily cases per million, daily deaths per million, and the effective reproduction rate were calculated to measure the effectiveness of COVID-19 vaccine policies implementation in each of these three countries. (3) Results: Italy, India, and South Africa differ in the start date of COVID-19 vaccination, vaccine types, vaccine appointments, and whether vaccinations are free. The COVID-19 vaccination rates in these three countries varied widely, with Italy having the highest and South Africa the lowest. COVID-19 vaccination has had a positive effect on reducing daily deaths and stabilizing the effective reproduction rate. The three countries had experienced more than one outbreak spike due to the spread of new mutated strains since the start of COVID-19 vaccination. (4) Conclusions: This study concluded that responding to the COVID-19 pandemic requires active promotion of basic and booster vaccinations to comprehensively build up the population immune barrier. Promoting equitable distribution of COVID-19 vaccine internationally and solidarity and cooperation among countries maximizes global common interests. By combining vaccination with non-pharmaceutical interventions, the pandemic can be prevented and controlled comprehensively and systematically in three aspects: detection of the source of infection, reduction of transmission routes, and protection of susceptible populations.

## 1. Introduction

On 31 December 2019, the first confirmed case of coronavirus disease 2019 (COVID-19) caused by SARS coronavirus-2 (SARS-CoV-2) was reported in Wuhan, China. On 31 January 2020, the World Health Organization (WHO) declared COVID-19 a global “pandemic” and classified it as a public health emergency of international concern [1]. As of 1 April 2022, COVID-19 has caused more than 490 million confirmed cases and more than 6 million deaths worldwide, and has placed a heavy burden on national healthcare systems and the world economy [2]. The WHO survey reports that the COVID-19 outbreak has affected critical health services in 90% of countries worldwide, discontinuing many routine and elective services in most countries, and forcing the suspension of critical services such as cancer screening treatment and HIV treatment in low-income countries [3]. At the same time, the COVID-19 pandemic also had a strong impact on the world economy, resulting in a huge loss of global economic output.

Since the beginning of the pandemic, many governments have actively developed policies to contain the spread of the virus and mitigate the negative effects of COVID-19. Along with the successful development, evaluation, and production of multiple COVID-19 vaccines, the current COVID-19 vaccination has gradually become the basic approach for countries to deal with this pandemic. On 31 December 2020, the BNT162 COVID-19 vaccine, developed by Pfizer and BioNTech, was the first to receive an emergency use authorization (EUA) from the WHO to begin expanded supply and global distribution. Since then, the COVID-19 vaccine has been developed and produced worldwide at an unprecedented rate. Currently, a total of 10 COVID-19 vaccines have received EUA and are widely used, mainly developed and produced by Pfizer & BioNTech, AstraZeneca, Johnson & Johnson, Moderna, Novavax, Bharat Biotech, Sinovac Biotech, Beijing Institute of Biological Products, and other companies. The main types of vaccines are live attenuated, inactivated, protein-based, nucleic acid, and viral vector-based. Each type of vaccine has a subtle structure and advantages and disadvantages with respect to immunogenicity, safety, ease of use, and effectiveness [4].

Vaccination is an effective means of controlling the pandemic and reducing the risk of it. Promoting comprehensive vaccination and protecting vulnerable populations is an important part of the fight against the COVID-19 pandemic [5]. By increasing vaccination rates, the number of infection can be reduced and the spread of the virus interrupted, thereby reducing the social and economic burden of the disease. As of 1 April 2022, more than 10 billion doses of COVID-19 vaccine have been administered worldwide and more than 60% of the world population has received at least one dose of COVID-19 vaccine. The country with the highest COVID-19 vaccination rates is the United Arab Emirates, where 98.99% of the population has received at least one dose of COVID-19 vaccine. However, with only about 15% of the population in low-income countries having received at least one dose of COVID-19 vaccine, there are clear disparities in global vaccination rates and significant imbalances in vaccine distribution.

In this study, Italy, India, and South Africa were selected based on their level of COVID-19 vaccination, the pandemic situation, national characteristics, and the main limiting factors of COVID-19 prevention and control. Italy currently has the highest COVID-19 vaccination rates in Europe and is one of the few countries in Europe to adopt mandatory vaccination. However, the population is aging, so it was once the country with the highest COVID-19 mortality rate. India is the largest producer of COVID-19 vaccine in the world, but less than 60% of the population is fully vaccinated and there had been repeated shortages of the COVID-19 vaccine. India also faces a large gap between rich and poor, overcrowding, and the lack of medical resources within the country. As the birthplace of Delta virus, India once had the highest number of confirmed cases of COVID-19 in the world. South Africa has the highest COVID-19 vaccination rates in Africa, but remains well below the world average. South Africa is not only limited by the shortage of medical resources and scattered sources of vaccines, but also by the emergence of multiple COVID-19 variant strains and Omicron strains. By collating the COVID-19 vaccine policies of Italy, India, and South Africa, this study analyzed and compared the basic situation and effectiveness of vaccination in these three countries, with a view to providing guidance for COVID-19 vaccination in other countries, advancing the global vaccination progress, thus solving the COVID-19 pandemic prevention and control challenges as early as possible and mitigating the impact of the pandemic on social and economic life.

## 2. Methods

### 2.1. Data Collection

The COVID-19 epidemiological data used in this study were obtained from the Johns Hopkins University & Medicine Coronavirus Resource Center, Our World in Data Website, and the official websites of the national health ministries of Italy, India, and South Africa. The data were selected for the period from the start of COVID-19 vaccination in each of the three countries to 1 April 2022. Total vaccinations, COVID-19 vaccination rates, rates of fully vaccinated, rates of booster-vaccinated, and total confirmed cases were selected for cross-sectional comparison of COVID-19 vaccination in these three countries. Daily cases per million, daily deaths per million, and the effective reproduction rate (Rt) were calculated to measure the effectiveness of COVID-19 vaccine policies implementation in each of these three countries.

Total vaccinations are the sum of the basic and booster doses of the COVID-19 vaccine and are the total number of doses of COVID-19 vaccine administered. COVID-19 vaccination rates are the proportion of the population that has received at least one dose of COVID-19 vaccine, including the proportion of the population that is fully vaccinated and the proportion of the population that is partially vaccinated. Daily cases per million is the number of new confirmed cases per million population per day for each country. Daily deaths per million is the number of new deaths per million population per day for each country. The effective reproduction rate is an indicator of how many people are infected by one individual. If Rt = 2, this implies that each infected person transmits the virus to two others, resulting in an uncontrolled epidemic [6]. Rt can assess the current dynamics of infectious disease transmission in a timely manner, reflecting the severity of the pandemic, may be influenced by policy and population immunity levels. Achieving a value of Rt < 1 is a necessary condition to stop the spread of virus [7].

### 2.2. Policies Information

COVID-19 vaccine policies for Italy, India, and South Africa, all from official WHO documents or survey reports and official government websites of these three countries, including Ministero della Salute, Agenzia Italiana del Farmaco (AIFA), the Ministry of Health and Family Welfare of the Government of India, and the Department of Health of the Republic of South Africa. The COVID-19 vaccine policies collated for this study covered the following aspects: basic vaccination plan, vaccine development and supply, vaccine appointments, free vaccination or not, vaccination boosters, vaccination of minors, compulsory vaccination, vaccines procurement, expanding the vaccination population, incentives for vaccination, and vaccination support policies.

Finally, we conducted a cross-sectional comparison of the basic information on COVID-19 vaccination in Italy, India, and South Africa, summarized the COVID-19 vaccine policies for each of these three countries. Meanwhile, we plotted the epidemiological curves for each of the three countries and marked the main vaccination policies to assess the effectiveness of the COVID-19 vaccine policies adopted by each country.

## 3. Results

### 3.1. Basic Information on COVID-19 Vaccination in the Three Countries

Table 1 shows that the basic profile of COVID-19 vaccination in Italy, India, and South Africa have differed significantly. In terms of initiating vaccinations, Italy was the first to start mass COVID-19 vaccination, India started half a month later, and South Africa was the last to start vaccination due to vaccine procurement and other constraints. In addition, the COVID-19 vaccine administered in Italy and South Africa were both from Pfizer/BioNTech and Johnson & Johnson, while the COVID-19 vaccine administered in India was mainly locally developed or collaboratively developed. Compared to Italy and India, South Africa had more liberal COVID-19 vaccination policies, with no vaccine appointments required and free vaccination available to all citizens. Both Italy and India had phased in vaccine appointments and free vaccinations. Initially, in Italy, the COVID-19 vaccine was reserved by age group and was free of charge only for citizens within the specified age group. At a later stage, as vaccination rates increased and vaccine supplies became more available, the requirement to make appointments for the vaccine was abolished and free vaccination was introduced. In India, vaccine appointments were not required in the early stages and universal free vaccination was not introduced. Later on, with the reform of the COVID-19 vaccination policies, vaccination appointments, and free vaccination for adults were introduced. As of 1 April 2022, the total COVID-19 vaccination was 136.00 million in Italy, 18.4 billion in India, and 33.74 million in South Africa. Of these, India had the most and South Africa the least.

**Figure 1:**
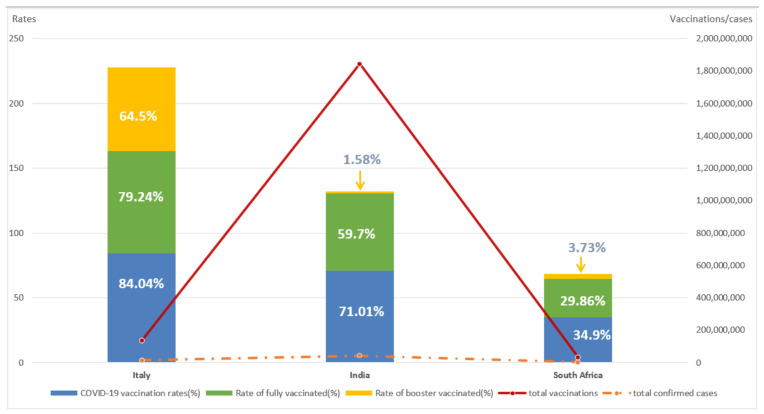
Total confirmed cases and COVID-19 vaccination rates in the three countries. Note: main axis refers to COVID-19 vaccination rates and rate of fully/booster vaccinated (left). Secondary axis refers to total confirmed cases and total vaccinations (right).

As can be seen through Figure 1, Italy had the highest level of COVID-19 vaccination of the three countries. Although total vaccinations was lower than that in India, COVID-19 vaccination rates, rates of fully vaccinated, and rates of booster-vaccinated were the highest in the three countries, at 84.4%, 79.24%, and 64.5%, respectively. India had the highest total vaccinations of the three countries, but both COVID-19 vaccination rates and rate of fully vaccinated were lower than in Italy, at 71.02% and 59.7%, respectively. Rate of people with booster vaccinated in South Africa was 3.73%, higher than India. However, COVID-19 vaccination rates and rate of fully vaccinated in South Africa were 34.9% and 29.86%, respectively, the lowest in these three countries. This related to the availability of vaccines, the level of primary health care and the level of national income in South Africa. Total confirmed cases in South Africa was around 3.7 million, while India and Italy had 43 million and 14 million, respectively. There was a wide disparity in total confirmed cases between the three countries, with South Africa having the fewest and India having the most.

### 3.2. Core COVID-19 Vaccine Policies of the Three Countries

#### 3.2.1. Italy

Italy launched a large-scale COVID-19 vaccination on 27 December 2020, and as of late May 2021 COVID-19 vaccination rates had exceeded 60%. One of the reasons that Italy had the highest COVID-19 mortality rate in Europe in the first wave of the pandemic was the severe ageing of the population. For this reason, Italy had given priority to vaccinating the elderly population with COVID-19, with the exception of workers in special positions, in order to reduce the risk of the virus in the elderly population. The supply of COVID-19 vaccine in the country was mainly distributed by the European Union (EU) and there was a tight supply of vaccine in the early stages of vaccination. However, as global vaccine production progressed, the tight supply of vaccine in Italy was gradually reduced. In April 2021, as the third wave of COVID-19 swept across Europe, countries across Europe began to explore more effective prevention measures to better respond to the worsening pandemic. At this time, Italy introduced the compulsory COVID-19 vaccination decree and gradually expanded the compulsory vaccination population and increased penalties as a way to increase COVID-19 vaccine coverage and flatten the epidemiological curve.

Italy was the first country in Europe to introduce mandatory COVID-19 vaccination. In line with the vaccine policies, the Italian government introduced the “Green Pass” policy, which was expanded as the pandemic evolved. The “Green Pass” was a powerful catalyst for vaccination in Italy and a scientific reference for other countries in Europe to increase their COVID-19 vaccination rates. Table 2 shows the main COVID-19 vaccine policies adopted in Italy.

#### 3.2.2. India

India officially started COVID-19 vaccination in mid-January 2021, the basic vaccination plan identified special post workers and high clinical risk groups as priority groups for COVID-19 vaccination in the country. India has a strong vaccine production and supply capacity and is an important global producer of COVID-19 vaccine; 60% of the vaccines supplied by the United Nations International Children’s Emergency Fund (UNICEF) to countries around the world were produced in India. The Serum Institute of India (SII), which had the highest global vaccine production capacity prior to the pandemic, also supplied a large quantity of COVID-19 vaccine to the world. However, nearly two-thirds of the COVID-19 vaccine produced in India was exported and the country was once in severe vaccine shortage. A second wave of the COVID-19 emerged in India in early April 2021, with high morbidity and mortality rates. As a result, after exporting some 66 million doses of vaccine to nearly 100 countries, India banned vaccine exports in mid-April 2021 to prioritize domestic demand. At the same time, India was unable to complete the basic vaccination program for citizens over 45 years of age at the time specified in the program due to severe vaccine shortages and had to postpone the vaccination program. In June 2021, as vaccine stocks grew, India revised the COVID-19 vaccine policies and began offering vaccinations free of charge to all adult citizens. The federal government had taken over the procurement of vaccines directly from the state governments. However, due to the introduction of online vaccine appointments in India in April 2021, many people in rural areas who did not know how to make an online appointment and who did not have access to vaccine appointment information would not be able to make an appointment. This has indirectly led to unsatisfactory COVID-19 vaccination rates in India. Table 2 shows the main COVID-19 vaccine policies adopted in India.

#### 3.2.3. South Africa

South Africa received the procured Oxford–AstraZeneca vaccine in mid-February 2021 and initiated the COVID-19 vaccination. However, a study found that the AstraZeneca COVID-19 vaccine may not be effective in preventing the spread of the new mutant virus B.1.351 in South Africa, so South Africa stopped administering the vaccine in mid-February 2021 and resold one million doses of the vaccine to other AU countries in April 2021. From the second half of 2021, the issue of vaccine supply in South Africa was gradually resolved and vaccination coverage was expanded, with South Africa starting to offer free vaccination against the COVID-19 to all adults. However, the lack of willingness of the population to be vaccinated has become a new obstacle. As a result, under the leadership of the President, South Africa launched a “Vaccination Weekend” and took a number of steps to motivate people to get vaccinated against COVID-19 in order to protect them from the serious complications of the COVID-19 virus. Since January 2022, several large South African companies implemented a mandatory COVID-19 vaccination policy for corporate employees. Although South Africa was actively promoting vaccination in the country in a number of ways, the growth of COVID-19 vaccination rates was slow and far below the world average due to poor basic health care, inadequate vaccination, and transportation facilities, and the country’s low income level. Table 2 shows the main COVID-19 vaccine policies adopted in South Africa.

### 3.3. The Effectiveness of COVID-19 Vaccination in These Three Countries

#### 3.3.1. Italy

As can be seen by the COVID-19 epidemiological curve in Italy, from the start of mass vaccination with COVID-19 at the end of December 2020 until 1 April 2022, Italy experienced two peak outbreaks from February to April 2021 and from December 2021 to March 2022, respectively. The second peak of the pandemic saw a sharp increase in daily new cases per million, reaching a record high of 377,906. However, daily deaths per million were significantly lower than the first peak of the pandemic at the beginning of the vaccination period. The maximum value of daily deaths per million was 11,894 at the first peak and dropped to 7.753 at the second peak. At the peak of the COVID-19 pandemic from February to April 2021, the average age of patients who died in Italy was 81 years, 66% of deaths had more than three underlying diseases, 18.6% of decedents had two underlying diseases, and 12.1% had at least one underlying disease. The number of deaths in the second wave of the pandemic peak decreased significantly as Italy had largely completed vaccination of high clinical risk groups, such as the chronically ill and the over-60 age group, in June 2021. In addition, Rt had remained stable below 1 after experiencing a small peak from January to April 2021. However, after the Delta variant replaced the Alpha variant in July 2021 Rt increased sharply to a higher level and continued until late January 2022. Of these, the booster dose of COVID-19 vaccine was initiated in Italy in mid-September 2021, followed by a month in which the Rt remained below 1 and the outbreak was controlled. In late October 2021, as the Omicron variant strain emerged and spread, Rt increased rapidly and exceeded 2 by the end of 2021, indicating that 1 COVID-19 infected person was able to trigger 2 cases of secondary infection at that stage.

**Figure 2:**
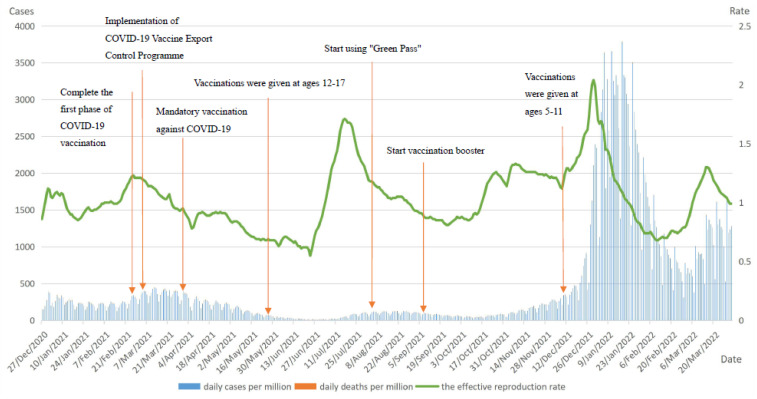
Curves of daily cases per million, daily deaths per million, and the effective reproduction rate in Italy. Note: main axis refers to daily cases and deaths per million (left). Secondary axis refers to effective reproduction rate (right).

In general, COVID-19 vaccination in Italy played significant roles in reducing daily deaths per million. However, new outbreaks were still occurring in Italy due to the emergence of mutated strains and their increasing infectivity. Figure 2 shows the curves of Italy daily cases per million, daily deaths per million, and the effective reproduction rate (Rt).

#### 3.3.2. India

In India, after the initiation of COVID-19 vaccination, two peak outbreaks occurred in daily cases per million from early April 2021 to mid-June 2021 and from early January 2022 to mid-February 2022, respectively. The second peak was more pronounced, shorter in duration, lower in daily cases per million, and quicker to fall back. Daily deaths per million showed only one significant peak between mid-April 2021 and the end of June 2021. Although India had completed the first and second phases of the COVID-19 vaccination plan by May 2021 and started the third phase of vaccination in the same month, due to the complete deregulation of the pandemic during this period, the emergence and spread of the more infectious B.1.617.2 Delta variant in India and the extreme shortage of vaccines in the country led to a sharp increase in the number of daily cases. India became the first country in the world to have more than 400,000 daily cases. In early 2022, with the global spread of the Omicron mutant strain, India saw a second peak of daily cases per million. The rapid increase in the number of confirmed cases caused the burden on the Indian healthcare system to become extremely heavy in the short term. The extreme shortage of medical supplies such as oxygen and ventilators, the severe shortage of medical staff, and the high rate of infection resulted in the death of critically ill patients who were admitted to the hospital without timely and effective treatment. People who were not hospitalized were not able to be vaccinated to reduce the risk of infection in the midst of a severe vaccine shortage in India, and were not 100% tested and diagnosed with COVID-19 infection, delaying treatment and leading to death. These two factors together led to the peak of daily deaths. In addition, the fluctuations in Rt were similar to those of daily cases per million, with the difference that the more infectious Delta variant led to higher Rt values in the second peak of the pandemic, with a 1.5-fold increase in the maximum value compared to the first wave.

**Figure 3:**
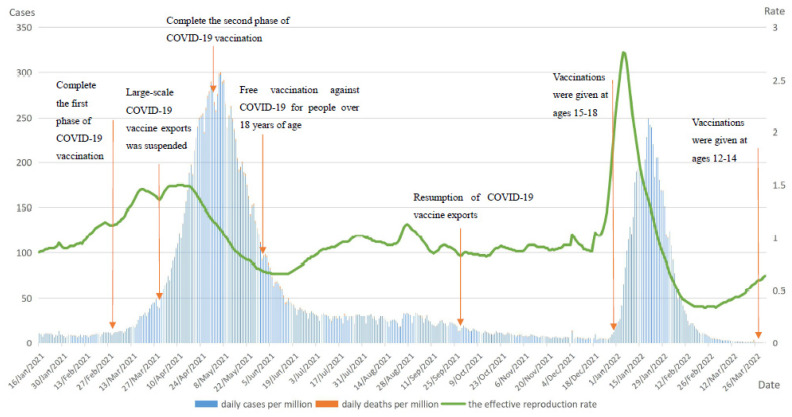
Curves of daily cases per million, daily deaths per million and the effective reproduction rate in India. Note: main axis refers to daily cases and deaths per million (left). Secondary axis refers to effective reproduction rate (right).

It is easy to see from the development of COVID-19 in India that even when COVID-19 vaccination was initiated, the emergence and spread of new strains still led to a rapid increase in the number of confirmed cases and deaths, reinforcing the severity of the pandemic. Figure 3 shows the curves of India daily cases per million, daily deaths per million, and the effective reproduction rate (Rt).

#### 3.3.3. South Africa

After the initiation of COVID-19 vaccination in South Africa in mid-February 2021, the first peak of the daily cases per million curve occurred between early June and late July 2021, reaching a peak of 441,108, a 31% increase compared to the peak of the pandemic before vaccination. It was the start of the spread of the highly infectious Delta strain in South Africa that first led to an increase in confirmed cases. Secondly, at this time, South Africa was in the second phase of COVID-19 vaccination and had only largely completed vaccination of health workers in the country, with full immunity not yet established in the population. In addition, because the pandemic situation had previously eased, control of the COVID-19 in South Africa was lax and the numerous public gatherings directly contributed to the surge in the number of infections. In late August 2021, the number of daily new cases per million began to decline in South Africa from the start of free vaccination for all adults. Between early December 2021 and early January 2022, with the spread of the Omicron mutant strain, a second peak in South Africa occurred, with a sharp increase in daily cases per million, reaching a maximum of 630,808. The duration of this peak was relatively short and the number of infected cases showed a sharp increase followed by a sharp decrease. When the situation eased, the South African government launched a vaccination booster to further enhance the population’s immunity to the virus.

**Figure 4:**
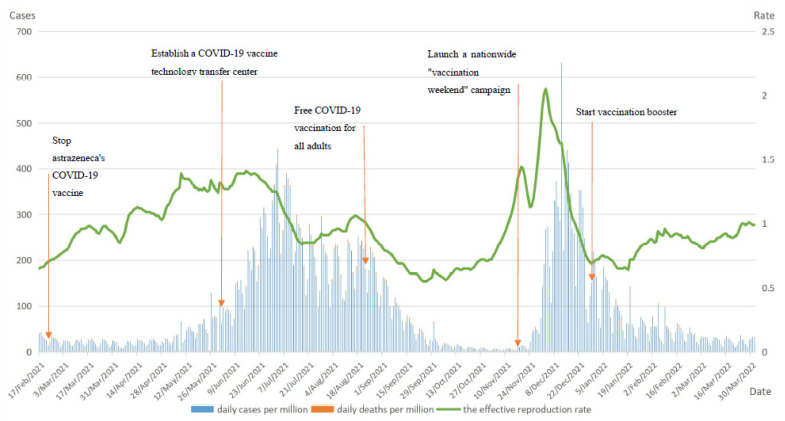
Curves of daily cases per million, daily deaths per million and the effective reproduction rate in South Africa. Note: main axis refers to daily cases and deaths per million (left). Secondary axis refers to effective reproduction rate (right).

There were two significant peaks in daily deaths per million in South Africa, which largely coincided with the timing of the peak in daily cases per million. The overall lower value of the second wave of peaks showed that the booster vaccination had a positive effect on the prevention of deaths. Rt in South Africa fluctuated more markedly with the implementation of COVID-19 vaccination. In late August 2021 South Africa started free COVID-19 vaccination for all adults, and the Rt remained below 1 for the next 2 months. The vaccine booster was started at the end of 2021 and the Rt was less than 1 until 1 April 2022, with minor fluctuations during this period but at manageable levels overall. Figure 4 shows the curves of South Africa daily cases per million, daily deaths per million, and the effective reproduction rate (Rt).

## 4. Discussion

This study confirms the positive effect of COVID-19 vaccination on reducing deaths and protecting susceptible populations by analyzing COVID-19 vaccine policies and their effects in Italy, India, and South Africa. Meanwhile, the difference in COVID-19 vaccination rates between these three countries is a direct reflection of the unequal global distribution of vaccines. We will further elaborate on the importance of COVID-19 vaccination for establishing global herd immunity, explore the necessity of promoting equitable distribution of vaccines, and discuss how to respond to the COVID-19 pandemic based on available research and epidemiological patterns.

### 4.1. Active Promotion of COVID-19 Vaccination Is Essential for the Full Construction of the Population Immunization

COVID-19 vaccination is currently a key public health strategy to reduce the overall burden of COVID-19 globally [8]. The epidemiological curves for Italy, India, and South Africa show that although all three countries experienced more than one spike in outbreaks caused by new mutant strains, there was a significant reduction in the number of deaths following COVID-19 vaccination. The WHO has proposed to achieve a global vaccination rate of 70% for COVID-19 in order to build a comprehensive immune barrier for the population. The current global vaccination rate is around 60%, which is still short of the WHO target.

To advance COVID-19 vaccination, it is important to continue to increase the proportion of the global population fully vaccinated on the one hand, and to continue to promote vaccine booster doses on the other. For countries with low COVID-19 vaccination rates, domestic vaccination efforts need to be promoted in line with WHO vaccination plans and national basic vaccination plan in order to reduce the number of COVID-19 hospitalizations, serious illnesses and deaths, provide adequate protection for the basic health of the population, and reach the global target of 70% complete vaccination coverage as soon as possible. For countries with a high level of completion of the COVID-19 vaccine basic dose, they should continue to promote the COVID-19 vaccine booster dose and accelerate the development of research and development for virus variants [9]. The Omicron variant is currently widespread worldwide; the large number of mutations it contains, its increased infectivity and immune escape compared to both Alpha and Delta strains, and its higher asymptomatic carrier rate may be major factors in the rapid global spread of this variant [10]. One study showed that the COVID-19 vaccine, while still effective in preventing hospitalization, severe illness, and death, showed a 41.4-fold decrease in neutralizing activity against the Omicron variant compared to the original strain [11]. Therefore, continuing to promote booster vaccinations worldwide is an important part of building up the population’s immune barrier as quickly as possible. Only by being adequately prepared can we respond to the mutation and evolution of the virus.

It is worth mentioning that promotion of COVID-19 vaccination requires increasing the willingness of the population to be vaccinated. Especially in many African countries, low willingness to vaccinate has caused delays in national vaccination efforts and slow depletion of vaccine stocks, which has led to a reduction in national vaccine procurement and a scaling back of production by vaccine manufacturers, ultimately affecting the adequate supply of vaccines. In addition, vaccine hesitation also affects COVID-19 vaccination. Vaccine hesitancy refers to delay in acceptance or refusal of safe vaccines despite availability of vaccination services [12]. COVID-19 vaccine hesitancy is a very common issue in high-income countries or regions. The countries with the highest vaccine hesitancy rates among high-income countries worldwide include the United Arab Emirates, the United States, Hong Kong, and Italy [13]. However, vaccine hesitancy is not only an issue in high-income countries, but is a complex, rapidly changing global problem that varies widely [12]. Vaccine hesitancy is generally associated with vaccine safety, negative perceptions due to rumors, distrust of health professionals or the health care system, and concerns about vaccine effectiveness [14]. Conspiracy beliefs are also significant factors influencing global COVID-19 vaccination. Coronavirus conspiracy beliefs were associated with long-standing individual prejudices, distrust of medical treatment, vaccine hesitancy, and vaccine decision making [15]. Conspiracy beliefs can contribute to medical mistrust, weaken social cohesion, and reduce COVID-19 vaccination. Moreover, conspiracy beliefs are strongly associated with vaccine hesitancy, which may reduce the confidence and motivation to vaccinate [16]. Therefore, to increase vaccination rates, promotion of COVID-19 vaccination also requires minimizing the level of vaccine hesitation among the public, adequately publicizing the safety and efficacy of vaccination, reducing the spread of misinformation, and incentives to increase the motivation of the population to vaccinate [17].

### 4.2. Promoting Equitable Global Distribution of COVID-19 Vaccine Is Critical

Currently, unequal distribution of COVID-19 vaccine remains a challenge in the global fight against the COVID-19 pandemic. The differences in COVID-19 vaccination rates and levels of vaccination shown in Italy, India, and South Africa are a microcosm of the inequitable distribution of vaccines globally. As of 1 April 2022, more than 10 billion doses of COVID-19 vaccine have been administered globally, and the proportion of the world’s population that has received at least one dose of COVID-19 vaccine is more than 60%. In low-income countries, however, the proportion is less than 20%. Of the 82 poorer countries in the world, only a few, such as Bangladesh, Cambodia, and Nepal, have achieved the target of 70% full COVID-19 vaccination [18]. While many middle- and high-income countries have started COVID-19 booster doses, poorer countries such as Somalia and Senegal have only single digit rates of COVID-19 fully vaccination.

The reasons for this inequitable distribution of COVID-19 vaccine between countries are complex. First, the gap between rich and poor countries is a major obstacle to the spread of vaccination, with high-income countries holding nearly 80% of the vaccine supply [19]. Second, there are also large gaps in countries’ capacity to provide universal access to the vaccine. Poor basic health care in low-income countries, inadequate support facilities for vaccine storage, transportation, and vaccination, weak health care systems, and the infestation of diseases such as AIDS and measles have also prevented governments from distributing COVID-19 vaccine to the population. Third, most poor countries have a fragmented and unstable source of COVID-19 vaccine. The main source of COVID-19 vaccine in these countries relies on donations from other countries, with much of Africa relying on donations from the COVID-19 Vaccine Global Access Facility (COVAX), as well as from China, India, and the United States.

Inequity in the distribution of COVID-19 vaccine between countries is more than a matter of equality, as this persistent inequity increases the risk of new mutant strains in populations with low COVID-19 vaccination rates. The emergence of Omicron highlights the need to guarantee the equitable distribution of vaccines and other medical resources [11]. Achieving equitable distribution of COVID-19 vaccine requires not only developing new vaccines, but also making them affordable, available, and effective [20]. The barrel theory states that the volume of water in a barrel depends on the shortest board in the barrel. That is, the least-developed element of something determines its overall level of development. Control of the global COVID-19 pandemic depends on control in the worst-case countries [21]. Therefore, countries need to work together to support vaccine multilateralism. Developing Countries Vaccine Manufacturers Network (DCVMN), Global Vaccine Action Plan (GVAP), COVID-19 Vaccine Global Access Facility (COVAX), and other international organizations should adopt a coordinated and collaborative approach to reinvigorate the response of developing countries to the COVID-19 pandemic [4]. A more equitable distribution of the COVID-19 vaccine would help to contain the pandemic earlier, reduce the risk of new mutant strains emerging, and contribute to world economic recovery and development, thereby maximizing the global common good [18].

### 4.3. Comprehensive Prevention and Control Measures Are Necessary

Until a vaccine was available, the main way to respond to COVID-19 was to contain or mitigate the spread of the pandemic through the implementation of non-pharmaceutical interventions. Non-pharmaceutical interventions (NPIs) are public health measures to reduce the spread of the virus by reducing the rate of exposure, such as contact tracing, international travel controls, closure of public transport, wearing of masks, closure of workplaces, cancellation of public events, etc. [22]. Italy, India, and South Africa all implemented a number of non-pharmacological interventions at the peak of the pandemic before and during the initial phase of COVID-19 vaccination. In Italy, at the first peak of the pandemic after the launch of mass vaccination, the country was divided into red prevention zones, people were banned from travelling across the region, and public places were closed at regular intervals. In India, the train pod hospital scheme was introduced, temporary isolation beds were set up, and domestic travel was banned, drawing on the experience of China’s square-cabin hospitals. In South Africa, regulations on wearing masks and maintaining social distance were put in place and lockdown measures were imposed.

COVID-19 vaccination aims to protect susceptible populations. However, responding to a global pandemic such as COVID-19 requires a concerted effort in three areas: identifying the sources of infection, reducing the routes of transmission, and protecting susceptible populations. Premature cancelling of non-pharmaceutical interventions before COVID-19 vaccination rates have reached a level sufficient to establish a solid population immunity barrier may result in the loss of hard-won control gains [23]. Therefore, the prevention and control of COVID-19 should be comprehensive and systematic, and multiple measures should be taken to develop an integrated strategy.

## 5. Conclusions

By analyzing COVID-19 vaccine policies and their implementation effects in Italy, India, and South Africa, this study confirmed that the vaccination had a positive effect on reducing the number of deaths and stabilizing Rt. This study also concluded that COVID-19 vaccination should be actively promoted, the proportion of the fully vaccinated global population should be continuously increased, booster doses should be promoted, and herd immunity should be established as soon as possible. It is also necessary to raise the willingness of the population to be vaccinated and to be aware of the influence of vaccine hesitation and conspiracy beliefs. Uneven levels of global COVID-19 vaccination and uneven distribution of the vaccines remain urgent issues to be addressed. Addressing these issues requires countries to work together and cooperate in solidarity to maximize global benefits. Promoting equitable distribution of COVID-19 vaccines is essential for the prevention and control of COVID-19. Vaccination aims to protect susceptible populations. Response to COVID-19 also requires identification of the source of infection and reduction of transmission routes. Comprehensive prevention and control is necessary.

## Figures and Tables

**Table 1:** Basic information on COVID-19 vaccination in Italy, India, and South Africa.

	Italy	India	South Africa
Start date of vaccination	27 December 2020	16 January 2021	17 February 2021
Vaccines administered	Pfizer/BioNTech, Moderna, AstraZeneca, Novavax, Johnson & Johnson	Covieshield, Covaxin, Sputnik V	Pfizer/BioNTech, Johnson & Johnson
Total vaccinations	136.00 million	18.4 billion	33.74 million
COVID-19 vaccination rates	84.4%	71.02%	34.9%
Rate of fully vaccinated	79.24%	59.7%	29.86%
Rate of booster vaccinated	64.5%	1.58%	3.73%
Vaccine appointment	Vaccine appointments were made online or by phone and are required by age group from January to June 2021.	Vaccine appointments were made online from 28 April 2021.	No appointment necessary for vaccination.
Free vaccination or not	Free vaccinations by age group.	Free vaccinations for all adults in public hospitals after June 2021. Private hospitals charge about 150 rubles for vaccination.	Free vaccination for the entire population.

Notes: the start date of vaccination reflects the local time in each country.

**Table 2:** COVID-19 vaccine policies of Italy, India, and South Africa.

	Aspects		Italy	India	South Africa
		Countries			
**Similarities**	**Basic vaccination** **plan**		Phase 1 vaccination: medical staff and health workers; residents and staff of nursing homes and orphanages; elderly group over 80 years old. Phase 2 vaccination: people at high clinical risk with underlying and chronic diseases; elderly people aged 60–79 years. Phase 3 vaccination: people aged 16 years and above who are not at particular risk. Phase 4 vaccination: people other than those in the above three phases.	Phase 1 vaccination: medical workers, police officers, and other front-line personnel in epidemic prevention and public officials. Phase 2 vaccination: people aged 40–59 with underlying diseases; elderly people aged 60 and above. Phase 3 vaccination: citizens aged 45 and above. Phase 4 vaccination: people in the age group of 18–44 years old. Phase 5 vaccination: people in the age group below 18 years old.	Phase 1 vaccination: over 1 million health care workers were the first to be vaccinated. Phase 2 vaccination: older age groups over 60 years old; people with comorbidities. Phase 3 vaccination: general population in the 50–59 age group. Phase 4 vaccination: general population in the 35–49 years age group. Phase 5 vaccination: age group 18–34 years old.
	**Vaccine** **development and supply**		In late August 2020, ReiThera, the COVID-19 vaccine developed in Italy, entered clinical trials. At the beginning of March 2021, the Italian government implemented the COVID-19 Vaccine Export Control Scheme to stop the export of the vaccine to Australia in order to bridge the supply gap. On 16 December 2021, the COVID-19 vaccine for children aged 5 to 11 years was approved and launched in Italy.	In January 2021, both the Covieshield vaccine, produced by SII in collaboration with AstraZeneca, and the indigenous Covaxin vaccine in India were developed and approved by the Indian drug regulatory authorities for use. At the end of January 2021, India started to provide vaccines to foreign countries in two forms: free of charge to neighboring and regional countries, commercial contracts. From April 2021, India suspended large-scale exports of COVID-19 vaccine to prioritize vaccination needs in the country. In April 2021, India gave urgent approval for the import and use of the Russian Sputnik V COVID-19 vaccine. In September 2021, the development of the ZyCoV-D COVID-19 vaccine in India was completed and approved for use in people aged 12 years and above. In October 2021, India resumed exports of COVID-19 vaccine, with priority supply to neighboring countries and the COVID-19 Vaccine Implementation Programme (COVAX).	In April 2021, South Africa reselled one million AstraZeneca COVID-19 vaccines to African Union (AU) countries. In late June 2021, led by the World Health Organization (WHO), South Africa established the first COVID-19 Vaccine Technology Transfer Centre to provide companies in low and middle income countries with the know-how and licences to produce the COVID-19 vaccine.
	**Vaccination of** **minors**		At the end of May 2021, Italy started offering the COVID-19 vaccination to the underage group aged 12–17 years. In mid-December 2021, Italy started vaccinating children aged 5 to 11 years with the new crown vaccine.	In early January 2022, India fully launched COVID-19 vaccination for the 15 to 18 year age group. The Indian Ministry of Health asked localities to set up specialized vaccination centers to prevent confusion with adult COVID-19 vaccination. In mid-March 2022, India started the COVID-19 vaccination for children aged 12–14 years.	On 10 September 2021, Kexin vaccine launched pediatric clinical trial in South Africa and inoculated first volunteer. On 20 October 2021, the South African government began offering the COVID-19 vaccination to children aged 12 years and above.
	**Vaccination boosters**		On 12 September 2021, the Italian Medicines Agency (AIFA) approved the first booster dose of the COVID-19 vaccine for people at high risk with weakened immune systems and for people over 80 years of age. In October 2021, Italy started the first booster dose of COVID-19 vaccine for groups aged 60 years and older who had completed the vaccination for more than 6 months. On 1 December 2021, the first booster dose of COVID-19 vaccine for the under-60 age group began. In mid-December 2021, the Italian government shortened the interval between the basic and booster doses of COVID-19 vaccine from 5 months to 4 months. On 12 April 2022, Italy started the second booster dose of COVID-19 vaccine for people over 80 years of age and people over 60 years of age who were at clinical risk.	In early January 2022, India began a booster dose of COVID-19 vaccine for healthcare workers, frontline workers and people over 60 years of age with underlying medical conditions.	In early November 2021, the South African government provided the first booster dose to health workers who have received the Johnson & Johnson vaccine. At the end of December 2021, South Africa began offering the first booster dose of the vaccine to people who had been fully vaccinated for more than 6 months. In mid-March 2022, the South African Department of Health announced the commencement of the second booster dose for people who had received the Johnson & Johnson vaccine.
**Differences**	**Compulsory vaccination**		At the beginning of April 2021, the COVID-19 vaccination was made compulsory for health professionals, health care workers and health care workers working in the public health system, private health care institutions, nursing homes, pharmacies, and specialized research centers. Italy provided for immunity from medical malpractice for medical personnel carrying out the national vaccination program for manslaughter and very serious personal injury caused by the administration of vaccines. Compulsory booster vaccination for Italian health workers and nursing home staff, including lay health workers, law enforcement personnel, army and military personnel, and all school staff in December 2021. In February 2022, Italy started mandatory vaccination against COVID-19 for the over-50 age group and imposed a fine of €100 for not completing the vaccination in time and for those who did not receive it.	No compulsory COVID-19 vaccination.	No compulsory COVID-19 vaccination.
	**Vaccination support policies**		In August 2021, the “Green Pass” was introduced in Italy to show the vaccination status of the population and the results of nucleic acid tests. Only people with a “Green Pass” could enter public places such as restaurants, bars, and cinemas. In September 2021, Italy extended the Green Pass to schools and long-distance public transport. On 15 October 2021, the Italian government made the use of the Green Pass compulsory in all workplaces. All employees in the public and private sectors were required to use the Green Pass for employment, and social workers were required to hold a Green Pass for employment. This is to prove that the employee had received at least one dose of COVID-19 vaccine, had tested negative for the virus or had recently recovered from COVID-19 infection.	No “Green Pass” was used.	No “Green Pass” was used.
	**Vaccines** **procurement**		The COVID-19 vaccines in Italy were procured centrally by the government.	In May 2021, India divided vaccination into three parts: federal hospitals, state hospitals, and private hospitals. Vaccine manufacturers sold half of the vaccine stock to the federal government, a quarter to state hospitals and private hospitals. People aged 18–44 years were required to purchase their own COVID-19 vaccine. In June 2021, the Federal Government of India started to take over the procurement of vaccine from the states. The federal government procures the COVID-19 vaccine directly from the vaccines manufacturer and distributes it to the states free of charge. Free vaccination for the COVID-19 vaccine for citizens of India over 18 years of age.	The COVID-19 vaccines in South Africa were procured centrally by the government.
	**Expand and incentivize vaccination**		No vouchers were given to people to incentivize vaccination.	No vouchers were given to people to incentivize vaccination.	In May 2021, South Africa began offering Pfizer vaccination to high-risk groups such as the elderly, people with chronic diseases and those working in basic industries. In late August 2021, South Africa began offering free COVID-19 vaccination to all adults. On 1 October 2021, South Africa launched a nationwide “Vaccination Weekend” to encourage people to get vaccinated. On 1 November 2021, R100 worth of vouchers were given to all adults over the age of 60 who had been vaccinated. At the same time, R500,000 in vouchers were given to the best performing vaccination sites in the country. From mid-November 2021, South Africa issued vouchers to all people in the 50–59 age group who received their first dose of vaccine.

## Data Availability

All data generated or analyzed during this study are included in this published article.
